# Machine-learning for the prediction of one-year seizure recurrence based on routine electroencephalography

**DOI:** 10.1038/s41598-023-39799-8

**Published:** 2023-08-04

**Authors:** Émile Lemoine, Denahin Toffa, Geneviève Pelletier-Mc Duff, An Qi Xu, Mezen Jemel, Jean-Daniel Tessier, Frédéric Lesage, Dang K. Nguyen, Elie Bou Assi

**Affiliations:** 1 Department of Neurosciences, Université de Montréal, Montréal, Qc Canada; 2 Institute of Biomedical Engineering, École Polytechnique de Montréal, Montréal, Qc Canada; 3 Centre de Recherche du CHUM (CRCHUM), Montréal, Qc Canada; 4 Centre de Recherche de l’institut de Cardiologie de Montréal, Montréal, Qc Canada

**Keywords:** Computational neuroscience, Epilepsy, Biomarkers

## Abstract

Predicting seizure recurrence risk is critical to the diagnosis and management of epilepsy. Routine electroencephalography (EEG) is a cornerstone of the estimation of seizure recurrence risk. However, EEG interpretation relies on the visual identification of interictal epileptiform discharges (IEDs) by neurologists, with limited sensitivity. Automated processing of EEG could increase its diagnostic yield and accessibility. The main objective was to develop a prediction model based on automated EEG processing to predict one-year seizure recurrence in patients undergoing routine EEG. We retrospectively selected a consecutive cohort of 517 patients undergoing routine EEG at our institution (training set) and a separate, temporally shifted cohort of 261 patients (testing set). We developed an automated processing pipeline to extract linear and non-linear features from the EEGs. We trained machine learning algorithms on multichannel EEG segments to predict one-year seizure recurrence. We evaluated the impact of IEDs and clinical confounders on performances and validated the performances on the testing set. The receiver operating characteristic area-under-the-curve for seizure recurrence after EEG in the testing set was 0.63 (95% CI 0.55–0.71). Predictions were still significantly above chance in EEGs with no IEDs. Our findings suggest that there are changes other than IEDs in the EEG signal embodying seizure propensity.

## Introduction

Epilepsy is a chronic neurological condition defined as an enduring, pathological propensity to recurring seizures^1^. Predicting the risk of seizure recurrence is at the heart of the diagnosis and management of people with epilepsy (PWE). The electroencephalogram (EEG), a 20- to 60-min recording of the electrical activity of the cerebral cortex via scalp electrodes, is a cornerstone of the estimation of seizure recurrence risk. The hallmark of epilepsy on the EEG is the interictal epileptiform discharge (IED): a brief, sharp discharge emanating from the background rhythm between seizures. In several clinical scenarios, such as after a first unprovoked seizure, before withdrawing antiseizure medication (ASM), and after surgical resection of an epileptic focus, visual identification of IEDs on routine EEG grossly doubles the risk that a patient will have seizure recurrence in the next years^2^. This impacts ASM management and prescription of ancillary tests.

Unfortunately, spikes are elusive: in PWE, a 20-min EEG captures spike in only 29–55% of cases^3,4^. As a result, the sensitivity of EEG for predicting seizure recurrence is limited^5^. In addition, IEDs are subject to overinterpretation with an inter-rater agreement that is only moderate, even among fellowship-trained neurophysiologists^6^. The overidentification of sharply contoured waveforms and normal variants as epileptiform can lead to the misdiagnosis of epilepsy, particularly in the event of a poor clinical history^7,8^. Finally, once the diagnosis of epilepsy is established, IEDs on routine EEG do not correlate well with disease activity, limiting their usefulness to monitor ASM therapy^9–12^. A biomarker of seizure propensity that is automated, objective, and independent of IEDs would heavily impact clinical practice by reducing diagnostic error, accelerating treatment in patients at high risk of seizures, avoiding the consequences of overdiagnosis in the others, and monitor disease activity.

Several studies have suggested that the routine EEG can capture non-visible anomalies in cortical activity in patients with both focal and generalized epilepsies^13–18^. These differences include subtle power shifts in specific frequency bands^19–22^, changes in regularity of the signal^23,24^, or presence of power scaling laws^25^. However, key questions were not addressed in previous studies, such as the reproducibility on external data and the impact of confounders like age and antiseizure therapy. In addition, previous studies are underpowered (with samples smaller than 100 patients)^26^. Thus, the potential predictive performances of computational EEG biomarkers in the clinical setting remain unknown^27^. There is a need for high-powered cohort studies to assess the diagnostic accuracy of these biomarkers and initiate their clinical translation.

In this paper, we develop and validate predictive models for the prediction of seizure recurrence at one year based on the computational extraction of biomarkers from the routine EEG signal. We train the model on a large retrospective cohort of consecutive patients undergoing routine EEG and validate the predictive accuracy on a temporally shifted cohort of patients. We investigate whether predictive accuracy is independent of IEDs and other clinical confounders.

## Methods

### Patient population and clinical file review

We retrospectively recruited all consecutive patients who underwent a routine EEG at the University of Montreal Hospital Center (CHUM) between January 2018 and June 2019. Routine EEGs (both awake and sleep recordings) recorded between January and December 2018 constituted the training set, while EEGs recorded between January and June 2019 constituted the held out testing set. We excluded EEGs with no follow-up visit available after the EEG, uncertain diagnosis at the end of the available follow-up, or with excessive artifacts or seizures (as per the EEG report). For the testing set, we additionally excluded patients for whom an EEG was already included in the training set. We reviewed the patients’ entire medical chart for clinical information: demographics (age, sex), comorbidities at the time of the EEG, epileptogenic factors, reason for EEG, presence of focal brain lesion on neuroimaging (when available), and number of ASMs. For PWE, we collected type of epilepsy, age of epilepsy onset, and date of the first seizure after the EEG. If the date of first seizure after the EEG was not available, we estimated it by linear interpolation based on the seizure frequency reported in the visit after the EEG. From the EEG report, we extracted the type of recording (awake or sleep deprived), deepest sleep stage achieved, presence of IED, and presence and degree of abnormal slowing. All clinical information was stored on a REDCap database hosted on the CHUM research center’s servers.

### Outcomes

The primary outcome is the patient-reported seizure recurrence during the first year of follow-up after the EEG, as provided in the medical notes. We considered only unprovoked seizures and auras, which include seizures that occurred in the setting of sleep deprivation and medication non-compliance^1^. The secondary outcome was the diagnosis of epilepsy, based on information available in medical notes by the appointed neurologist. The starting date of the diagnosis would be the date of the first seizure experienced by the patient. We only considered the diagnosis valid if it was concordant with International League Against Epilepsy criteria (Fisher et al. 2017): having had at least one seizure and either 1) a second seizure > 24 h apart or 2) an estimated risk of seizure recurrence ≥ 60% over the next 10 years^1^. If the diagnostic was not concordant, the patient would be excluded. The last outcome was active epilepsy: this required a diagnosis of epilepsy, at least one seizure in the year preceding the EEG, and seizure recurrence at any point after the EEG.

### EEG recording and processing

EEGs were recorded using a Nihon Kohden EEG system. The recording protocol is standardized based on national recommendations^28^. Awake EEGs were 20–30 min in duration and are recorded at 200 Hz through 19 electrodes arranged with the standard 10–20 distribution. They included two 90s periods of hyperventilation and photic stimulation from 4 to 22 Hz. Hyperventilation was not performed in patients > 80 y.o., in patients unable to cooperate with technologists, nor in patients with medical contraindications. Moreover, the patients were instructed to open or close their eyes at several times during the recording. Sleep deprived recordings were 60 min in duration, with the same activation procedures. An EEG technologist annotates the EEG in real-time. The protocol includes changes of montage at regular intervals during the recording, rotating through a total of seven montages. For this study, we converted the digital EEG to an average referential montage. EEGs were converted into EDF format and stored on the CHUM research center’s server for analysis.

**Figure 1:**
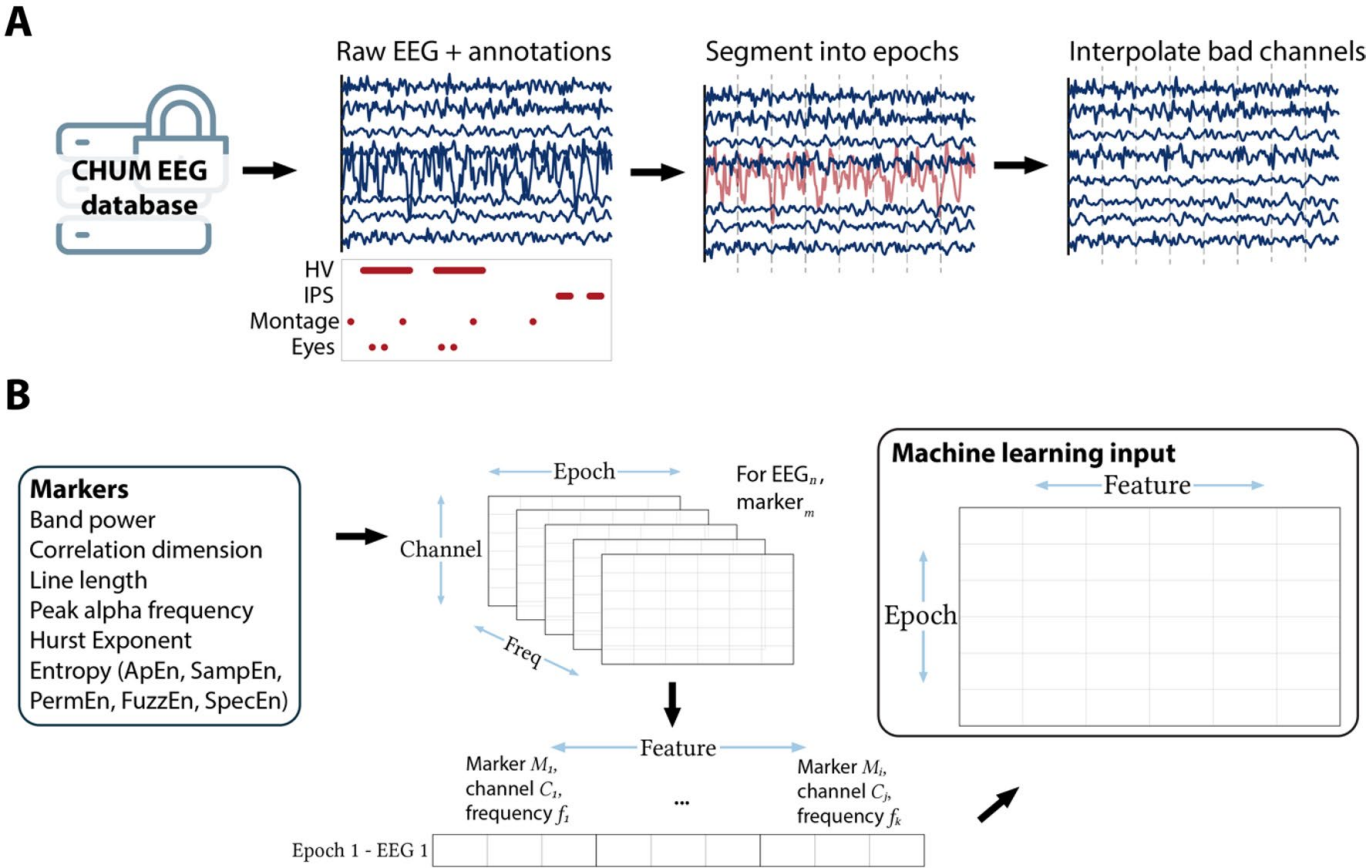
EEG processing and marker extraction methods. (**A**) Processing of a single EEG: extraction from the database in which the EEG is stored with annotations, segmentation into 10 s epochs according to pre-defined timepoints, identification of artefactual channels (in red) and interpolation. (**B**) Marker extraction: for each marker, one value is computed at each channel, epoch, and frequency bands. The values for a given epoch are used as input for the machine learning algorithm.

The processing pipeline is illustrated in Fig. 1A. EEG recordings were high pass filtered at 0.75 Hz and notch filtered at 60 Hz with a fast-impulse response (FIR) filter (hamming window). Ten-second epochs were extracted at pre-specified time points: every change of montage, every 15s during hyperventilation, every 15s for 2 min post-hyperventilation, every photic stimulation frequency, and every eye closure or opening. Artifact detection and interpolation were done using the *autoreject* algorithm^29^. For a given EEG recording, an optimal peak-to-peak amplitude threshold was found for each individual epoch/channel combination using fivefold cross-validation (CV). Rejected channels were interpolated using spherical splines. The preprocessing pipeline was written in Python (version 3.8) and is based on the *MNE* library.

### Extraction of computational markers

Ten univariate markers were extracted from the EEGs. The markers were selected based on previous literature, with the aim of capturing distinct linear and non-linear properties of the EEG signal across the time- and spectral-domain at each channel. The markers’ algorithms, mathematical details, and references are supplied in the Supplementary Method 1. Linear features were: band power (BP) in ten frequency bands (low [1–2 Hz] and high [2–4 Hz] delta, low [4–6 Hz] and high [6–8 Hz] theta, low [8–10 Hz] and high [10–13 Hz] alpha, low [13–20 Hz] and high [20–40 Hz] beta, low [40–75 Hz] and high [75–100 Hz] gamma), peak alpha frequency (PAF), and Hurst exponent (HE). For BP, the EEG were band pass filtered using a FIR (hamming window). Power spectrum density was calculated using a multitaper method, and the integral in each frequency band was estimated using Simpson’s method. For the PAF, the peak frequency of the alpha band (8–13 Hz, band pass filtered using a FIR window) was extracted for each EEG, epoch, and channel. HE was calculated for each EEG, epoch, channel, and wavelet decomposition level (see next paragraph), with a minimum window size of 10 points.

Non-linear features were: line length (LL), correlation dimension (CD), and five different entropy estimates: Approximate (ApEn), Sample (SampEn), Fuzzy (FuzzEn), Permutation (PermEn), and Spectral entropy (SpecEn). For non-linear features and for HE, one value was calculated for each feature, EEG, epoch, channel, and wavelet decomposition level (Fig. 1B**)**. The *Sym5* wavelet was used with six decomposition levels (with frequency range: 100–50 Hz, 50–25 Hz, 25–12.5 Hz, 12.5–6.25 Hz, 6.25–3.125 Hz, and 3.125–1.56 Hz)^30^. For entropies, optimal parameters were selected to maximize the inter-EEG vs. intra-EEG variance on five EEGs that were excluded from the study (*m* = 3, *r* = 0.25, *τ* = 5, *n* = 2, and *k* = 3). Missing values were imputed using multivariate iterative imputation. The calculation of the markers was independent on the outcomes.

### Machine learning model development and validation

The ML model’s task is to map the vector of linear and non-linear features’ values for a single EEG to a clinical outcome. The training was done epoch-wise—each EEG epoch fed as an independent learning observation. To prevent data leakage, epochs from the same patient were grouped together in the same CV split. The predictions for epochs of a single EEG were aggregated using the median to yield a single prediction per EEG. We also tested other percentiles (0.1–0.9 in 0.1 steps) for the aggregation of predictions (Supplementary Table S1). The clinical outcomes are described in the “Outcomes” section. The performance metric is the receiver operating characteristic area-under-the-curve (ROC AUC), selected for its robustness to class imbalance. Improvement over chance (IoC) was defined as an AUC significantly over 0.50.

Four ML algorithms were evaluated: Generalized linear model (GLM; *L*_1_- and *L*_2_-regularized logistic regression)^31^, support vector machine with radial basis function (SVM), random forest (RF), and gradient boosted trees (LightGBM)^32^. Supervised feature selection was performed with a linear *L*_1_-regularized SVM.

**Figure 2:**
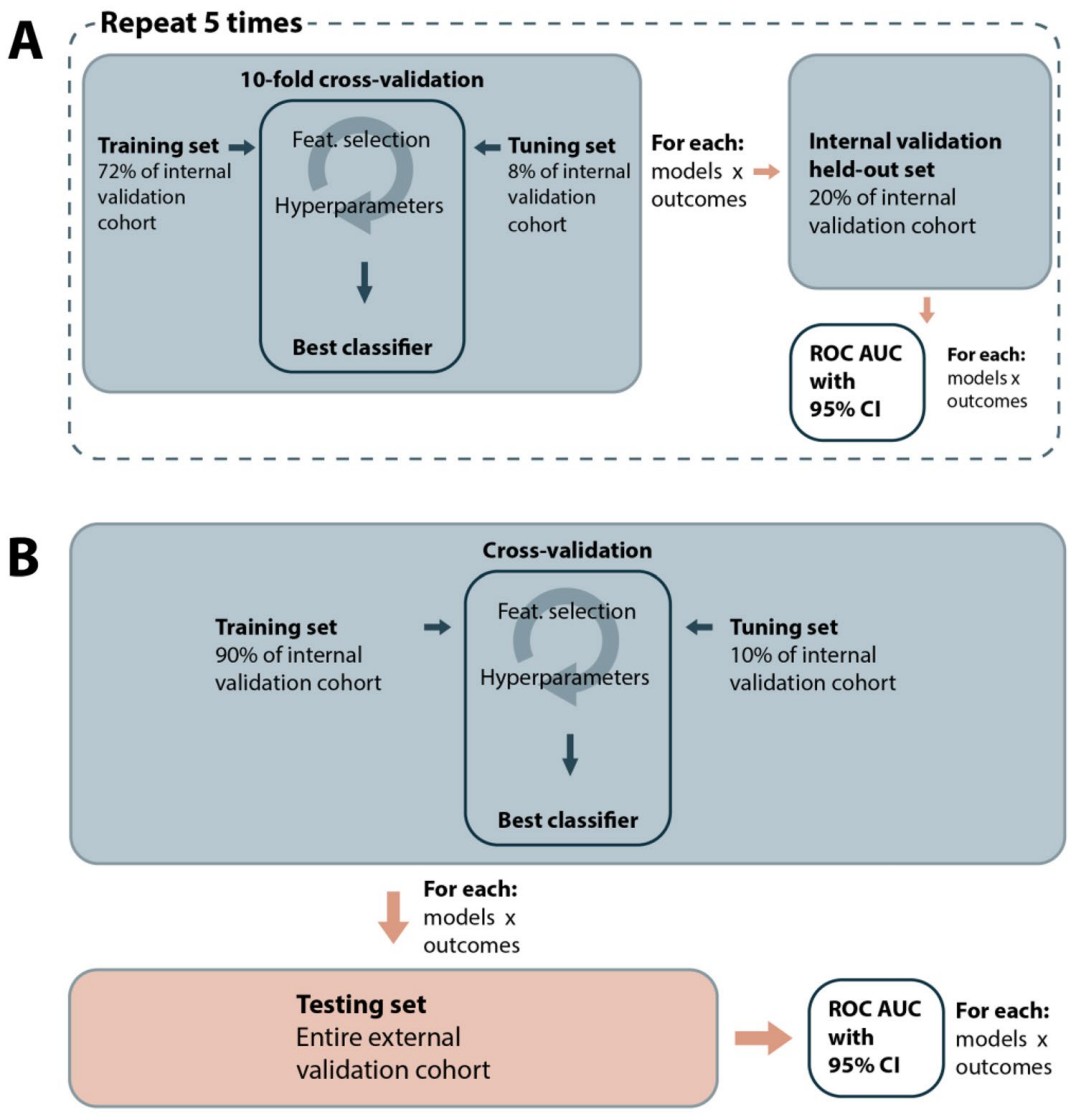
Machine learning evaluation methods. (**A**) Nested cross-validation with tenfold inner loop and fivefold outer loop. (**B**) Evaluation on the temporally shifted validation cohort (testing set). In blue: internal validation cohort (EEGs recorded in 2018). In red: testing cohort (EEGs recorded in 2019).

A nested CV was used first to evaluate the models, features, and clinical confounders on the training set (Fig. 2A). In nested CV, an inner-loop is used to optimize hyperparameters of the feature selection and learning model, and an outer-loop is used to validate the performances on separate data. It allows to estimate confidence intervals and is more robust than CV^33^. We used a tenfold CV for the inner-loop and a fivefold CV for the outer-loop, with patient-wise grouping of the EEG epochs at each level. ROC AUC values across outer-loop CV splits were averaged and 95% confidence intervals were estimated using LeDell’s curve based approach^34^. ROC AUC values were compared against the random classifier (ROC AUC = 0.50). Statistics were calculated at the EEG level (after aggregating predictions for all epochs in a single EEG). ML models were trained and validated using Python 3.8 (with classifiers from *Scikit-learn* and *LightGBM* libraries).

We tested the interacting effect of age, presence of IEDs, and presence of a focal lesion on neuroimaging to increase the performances of the algorithm. For age, we added interaction terms between scaled age and features to the set of features. For IEDs and focal lesions, we used a two-step classifier: first, if the factor is positive (e.g.: presence of IEDs), the model automatically outputs a positive prediction. If the factor is negative, the ML model’s predictions are used.

### Validation of predictive performances on testing set

We validated the performances of the best performing ML models on the testing cohort (Fig. 2B). First, we removed features that did not show IoC on the training cohort. Then, we performed a tenfold CV on the training data (EEGs from 2018) to select the best features and best hyperparameters for each of the four previously described models and three outcomes. The best feature-set/hyperparameters were used to train the models on the training data. The trained models were then applied to the testing set (EEGs from 2019) to emit probabilistic predictions. We computed the ROC AUC values from the probabilistic predictions, with 95% confidence intervals estimated by DeLong’s approach (single prediction by patient)^35^. For the primary outcome, we calculated the performance using only patients who had at least a one-year follow-up after the EEG. We also tested the outcomes on all testing patients (including those with follow-up shorter than one year). For comparison, we tested the classification performance of IED alone (presence vs. absence) and focal lesion alone (presence vs. absence) on the risk of seizure recurrence.

### Post-hoc analyses

Post-hoc analyses were performed on predictions from the LightGBM classifier. For each outcome, we evaluated the risk of bias of the classification for different subgroups by recomputing average AUC and 95% CI. The subgroups were age group (18–40, 40–60, and > 60 years old), sex, presence of focal lesion, presence of IED (absence, presence, and uncertain), presence of slowing, number of ASM (0, 1, ≥ 2), and epilepsy type (focal, generalized, and unknown). We investigated the performances in two specific subgroups: patient not yet diagnosed with epilepsy (undergoing evaluation for suspected seizures), and patients undergoing EEG pre-ASM withdrawal.

We also investigated the time-dependence of the predictions for seizure recurrence as well as the impact of clinical confounders using a multivariate survival model. We used the model’s predictions to separate the patients into a low-predicted risk and high-predicted risk (above vs. below average). We then fit a cox proportional hazard model to estimate the hazard ratio of seizure recurrence dependent on the model’s predictions, controlled for the following characteristics: age, sex, and number of ASMs (selected based on a directed acyclic graph presented in Supplementary Fig. S1). We checked the robustness of the choice of covariates with a sensitivity analysis (see Supplementary Method 2).

### Comparison of individual markers

We compared the predictions for seizure recurrence of individual markers between each other and with their combination. We repeated the nested CV independently for each marker, using only the values from this marker as input to the classification pipeline (keeping CV splits identical between markers).

### Sample-size estimation

Power analysis is described in Supplementary Method 3. With a significance level of 0.05, accounting for 12 multiple comparisons (3 outcomes × 4 models), we estimated that routine EEGs from a single year would provide us with a power > 0.9 for the expected effect size.

### Ethics

Ethics approval was provided by the CHUM Research Centre’s Research Ethics Board (Montreal, Canada, project number: 19.334). A waiver of informed consent was provided by the CHUM Research Centre’s Research Ethics Board due to the absence of diagnostic/therapeutic intervention and the absence of risk for the subjects involved. All methods were carried out in accordance with Canada’s Tri-Council Policy statement on Ethical Conduct for Research Involving Humans.

### Reporting standards

The reporting of the study conforms with the TRIPOD statement (Transparent reporting of multivariate prediction model for individual prognosis or diagnosis) when applicable^36^.

## Results

### Patient characteristics

Patients’ characteristics for the training cohort are described in Table 1. We screened 816 records for eligibility; 517 patients were included (549 EEG recordings). In this cohort, 132 EEGs were from patients who had seizure recurrence after the EEG (24%). There were 346 EEGs (63%) from PWE. The median age was 50 y.o. (IQR: 33–62 y.o.). Median follow-up after the EEG was 100 weeks (IQR: 42–135). In PWE, 248 EEGs (72%) did not show IEDs. The EEG was part of the initial evaluation of suspected seizure(s) in 286 cases (Supplementary Table S1).

**Table 1:** Description of the training (EEG recordings between January and December 2018) and testing cohorts (EEG recordings between January and June 2019).

	Training cohort (EEGs from 2018)		Testing cohort (EEGs from 2019)	
	Seizure freedom at one year	Seizure recurrence at one year	Seizure freedom at one year	Seizure recurrence at one year
Number of EEGs	417	132	217	84
Epilepsy diagnosis (%)	214 (51.3)	132 (100.0)	98 (45.2)	84 (100.0)
Age (median [IQR])	52.00 [35.00, 64.00]	37.00 [26.75, 55.00]	54.00 [36.00, 66.00]	37.00 [30.00, 57.25]
Sex = woman (%)	223 (53.5)	56 (42.4)	115 (53.0)	51 (60.7)
Total follow-up after eeg in weeks (median [IQR])	94.00 [35.00, 133.00]	136.00 [97.50, 163.25]	78.00 [32.00, 122.00]	123.50 [94.25, 141.00]
Epilepsy type (%)				
Focal	148 (35.5)	88 (66.7)	71 (32.7)	66 (78.6)
Generalized	56 (13.4)	41 (31.1)	21 (9.7)	13 (15.5)
No epilepsy	203 (48.7)	0 (0.0)	119 (54.8)	0 (0.0)
Unknown	10 (2.4)	3 (2.3)	6 (2.8)	5 (6.0)
Age of epilepsy onset (median [IQR])	24.00 [15.00, 43.00]	18.00 [13.00, 35.00]	28.00 [15.00, 53.00]	28.00 [14.00, 48.00]
Number of days since last seizure (median [IQR])	801.00 [225.00, 3525.00]	43.00 [9.50, 127.00]	613.00 [165.00, 1489.50]	32.50 [4.00, 90.00]
Number of ASM (%)				
0	197 (47.2)	11 (8.3)	108 (49.8)	19 (22.6)
1	149 (35.7)	58 (43.9)	80 (36.9)	31 (36.9)
2	48 (11.5)	45 (34.1)	21 (9.7)	15 (17.9)
3	19 (4.6)	13 (9.8)	6 (2.8)	14 (16.7)
4	4 (1.0)	5 (3.8)	2 (0.9)	3 (3.6)
5	0 (0.0)	0 (0.0)	0 (0.0)	2 (2.4)
Focal lesion on brain imaging (%)	142 (34.1)	53 (40.2)	76 (35.0)	44 (52.4)
Sleep deprived EEG (%)	51 (12.2)	13 (9.8)	33 (15.2)	18 (21.4)
IED (%)				
Absence	333 (79.9)	77 (58.3)	174 (80.2)	47 (56.0)
Presence	52 (12.5)	46 (34.8)	28 (12.9)	30 (35.7)
Uncertain	32 (7.7)	9 (6.8)	15 (6.9)	7 (8.3)
Abnormal slowing on EEG (%)	107 (25.7)	37 (28.0)	72 (33.2)	39 (46.4)

For the testing set, 429 records were screened for eligibility. After applying exclusion criteria, we included 301 EEGs from 261 patients (Table 1). The prevalence of seizure recurrence after EEG in this cohort was 32%. Other variables have a similar distribution to the training and validation cohort.

### Predictive performances on the internal validation cohort

**Figure 3:**
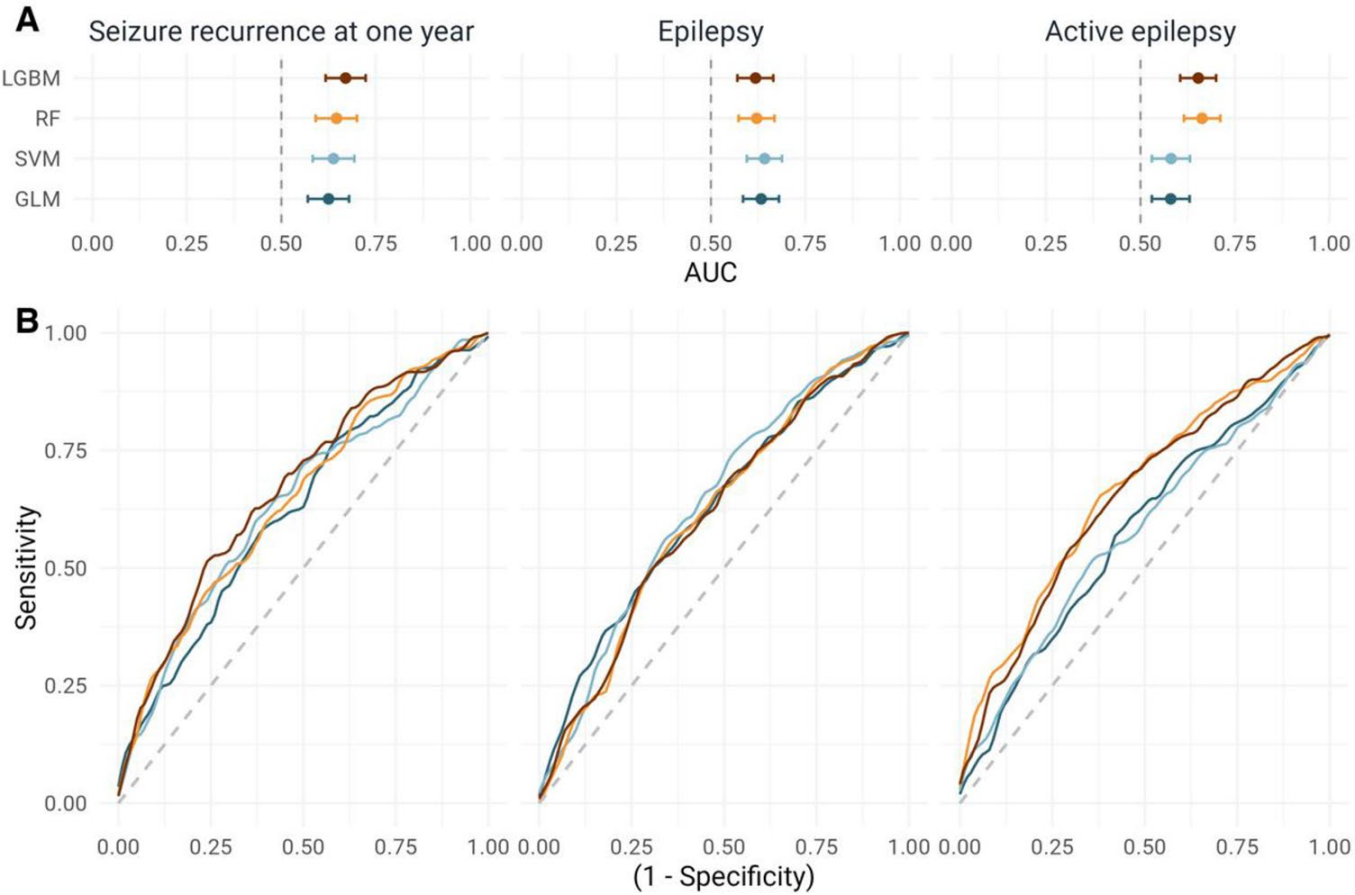
Classification performances for each classification algorithm and each clinical outcome. (**A**) AUC ROC with 95% interval estimated using nested five-fold cross-validation. (**B**) ROC curves for each algorithm.

For all outcomes, all four algorithms had statistically significant IoC (Fig. 3 and Table 2). For the prediction of seizure recurrence at one year, the best model was LightGBM with a ROC AUC of 0.67 (0.62–0.72). For the diagnosis of epilepsy, the best model was SVM with a ROC AUC of 0.64 (0.60–0.69). For active epilepsy, the best model was RandomForest with a ROC AUC of 0.66 (0.62–0.71). There was no statistical difference in performances across models for each outcome. The quantile used for aggregating predictions of the epochs from a single EEG had no significant impact (Supplementary Table S2).

**Table 2:** Classification performances on internal validation cohort estimated from nested CV.

Outcome	Classifier	AUC
Seizure recurrence at 1 year	GLM	0.62 (0.57–0.68)
	SVM	0.64 (0.58–0.69)
	RandomForest	0.65 (0.59–0.70)
	LightGBM	0.67 (0.62–0.72)
Epilepsy diagnosis	GLM	0.63 (0.58–0.68)
	SVM	0.64 (0.60–0.69)
	RandomForest	0.62 (0.57–0.67)
	LightGBM	0.62 (0.57–0.66)
Active epilepsy	GLM	0.58 (0.53–0.63)
	SVM	0.58 (0.53–0.63)
	RandomForest	0.66 (0.62–0.71)
	LightGBM	0.65 (0.60–0.70)

Adding clinical information to the feature set did not have a statistically significant effect. By integrating age, AUC was 0.67 (0.62–0.72). For the two-step model with IEDs, AUC was 0.66 (0.60–0.71) and for the two-step model with focal lesion, AUC was 0.58 (0.53–0.64).

### Survival analysis

The overall probability of remaining seizure free at one-year was 0.73 (95%CI: 0.69–0.77). When stratifying by the LightGBM model predictions, the one-year seizure free survival was 0.82 (0.78–0.87) in the high-predicted risk, and 0.61 (0.54–0.68) in the low predicted risk. In contrast, the one-year seizure free survival as a function of IEDs was 0.49 (0.40–0.61) in the presence of IEDs, and 0.78 (0.74–0.82) in the absence of IEDs.

**Figure 4:**
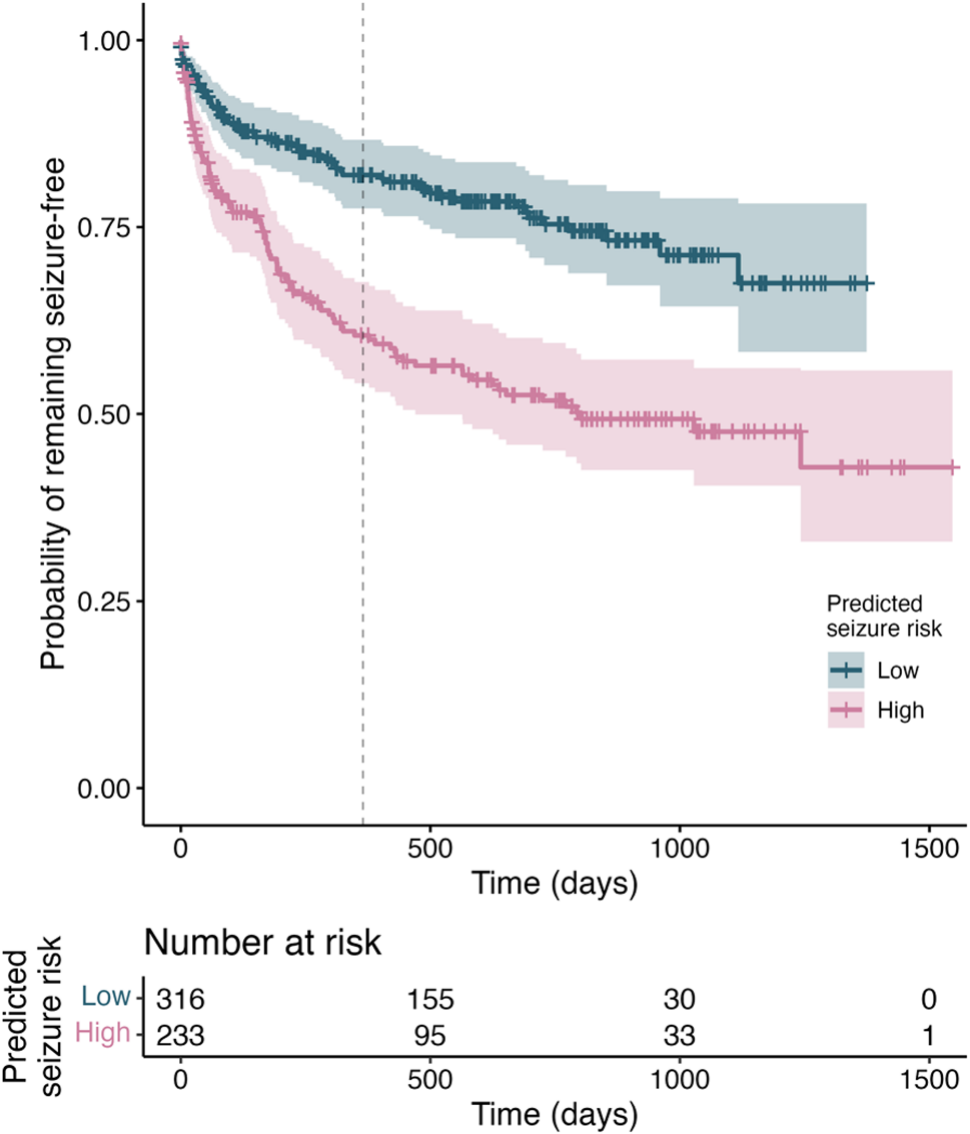
Survival curve for seizure freedom after the EEG, dependent on the risk predicted by the LightGBM model. Dashed line indicates one-year follow-up.

In the multivariate survival analysis, the adjusted hazard ratio of seizure recurrence for the model’s predictions was 1.22 (95%CI 1.07–1.40, *p* = 0.0029). The Kaplan–Meier curve (Fig. 4) shows separation between groups up to one year after the EEG. The risk of seizure recurrence was strongly associated with age (aHR: 0.68, 0.56–0.82, *p* < 0.001) and number of ASM (1.66, 1.43–1.92, *p* < 0.001). The sensitivity analysis showed robustness to different sets of covariates (Supplementary Method 2).

### Subgroup analyses

**Figure 5:**
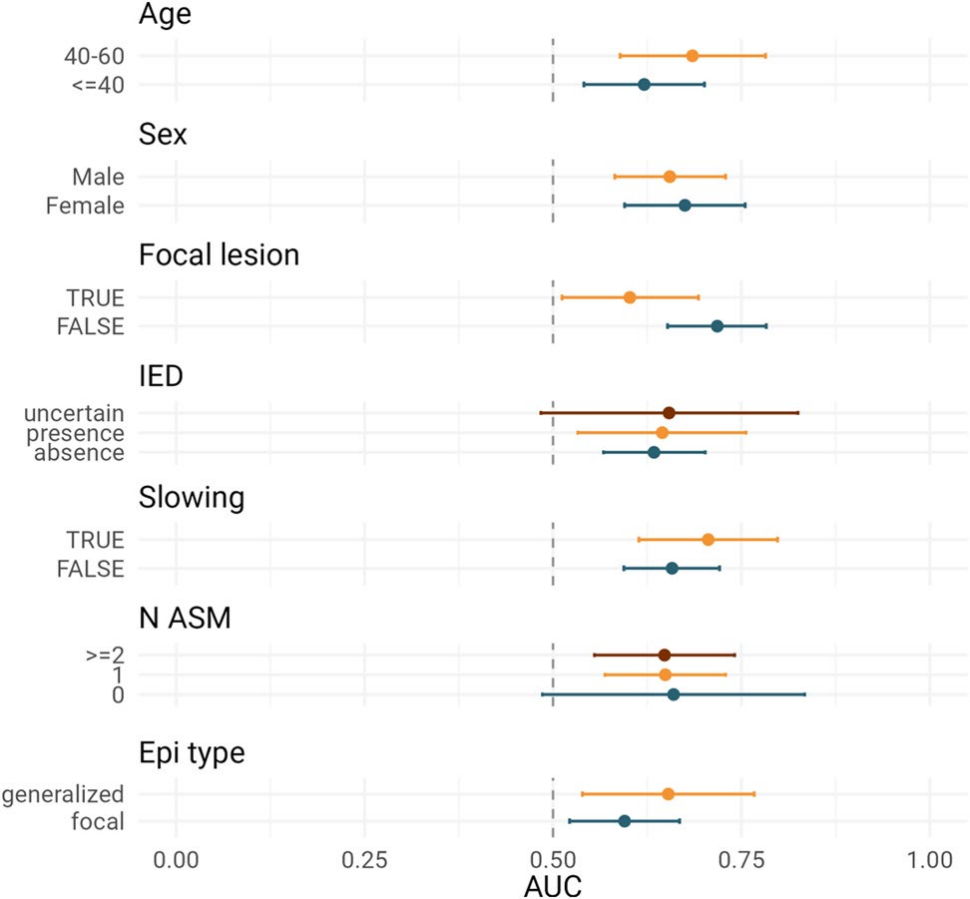
Predictive performances (ROC AUC) for the LightGBM model stratified by subgroups for each of the three outcomes, with 95% confidence intervals. The dotted line indicates AUC of 0.50. AUC ROC: Area-under-the-receiver operating characteristic curve.

In the subgroup analysis, there was no statistical differences between any strata for almost all outcomes (Fig. 5 and Supplementary Fig. S2). For seizure recurrence at 1 year almost all subgroups had performances that were significantly above chance. Only the absence vs. presence of focal lesion showed a trend towards increased AUC. For the epilepsy outcome, performances were not above chance for patients between 40 and 60 y.o. For some subgroups, sample size was small, and estimation were either not reliable (“uncertain” IEDs [all outcomes], no ASM [outcome “seizure recurrence”]) or impossible (patients > 60 y.o. [outcome “seizure recurrence”], ≥ 2 ASM [outcome “Epilepsy”]).

In the subgroup of patients undergoing initial evaluation for seizure(s) (N = 227), the ROC AUC was also statistically significantly above chance (0.65 [0.57–0.74]). For the subgroup of patients undergoing EEG before ASM withdrawal, AUC could not be estimated because of the small sample size (N = 32, < 1 per outcome in each nested CV fold).

### Comparison between markers

**Figure 6:**
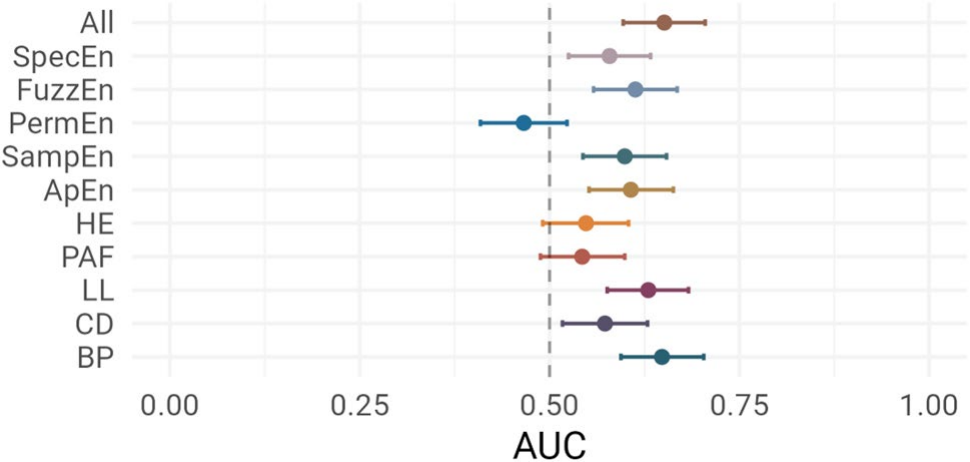
Comparison of predictive performances for all markers using a LightGBM model. *ApEn* Approximate entropy, *BP* Band power, *CD* Correlation dimension, *FuzzEn* Fuzzy entropy, *HE* Hurst exponent, *LL* Line length, *PAF* Peak alpha frequency, *PermEn* Permutation entropy, *SampEn* Sample entropy, *SpecEn* Spectral entropy.

The comparison between markers is shown in Fig. 6. The best markers were BP (AUC ROC 0.65 [95%CI: 0.59–0.70]), LL (0.63 [0.58–0.68]), and FuzzEn (0.61 [0.56–0.67]). PermEn, PAF, and SpecEn did not show IoC. The combination of all features had the greatest predictive performances (0.65 [0.60–0.70]).

We repeated the subgroup analysis for each individual feature (Supplementary Fig. S3). For age, relative performances of CD and SpecEn were decreased in patients 40–60 y.o., while for BP, they were decreased in patients ≤ 40 y.o. Presence of focal lesion decreased performances in all markers. Presence of IEDs decreased performance of all markers except BP and LL. The presence of abnormal slowing particularly reduced the performances of CD. In patients with one ASM, LL and BP had higher performances, while in patients with ≥ 2 ASM, only BP had IoC. The combination of markers reduced the impact of stratification, and was the only feature set that showed IoC in all strata.

### Validation on a temporally shifted cohort

In the testing cohort, the LightGBM model had IoC for predicting seizure recurrence at one year, with an AUC of 0.63 (95% CI 0.55–0.71). The performances on the entire testing cohort (including those with follow-up shorter than a year) were similar (0.64 [0.58–0.71]). The binary predictions had a negative predictive value (NPV) of 78%, a positive predictive value (PPV) of 37%, a sensitivity of 64%, and a specificity of 55%. In the absence of IEDs, the LightGBM predictions were still significantly above chance (seizure recurrence: 0.63 [0.55–0.71]). For comparison, in this cohort, IEDs (presence vs. absence/uncertain) had an AUC of 0.61 (0.56–0.69) for seizure recurrence at one year, while the presence of a focal lesion had an AUC of 0.59 (0.53–0.65). For the outcomes “epilepsy diagnosis” and “active epilepsy”, AUC for the LightGBM model was 0.64 (0.57–0.70) and 0.57 (0.50–0.63), respectively. We tested the two-step classifier with IEDs on the temporally shifted cohort for the prediction of seizure recurrence at one year, achieving an AUC was 0.70 (0.63–0.76). For the binary predictions, NPV was 80%, PPV was 51%, sensitivity was 83%, and specificity was 45%.

## Discussion

This study demonstrates that machine learning models trained on computational features automatically extracted from 20-min scalp EEG can predict seizure recurrence at one year with above-chance performances in a cohort of 778 consecutive patients undergoing routine EEG. The predictive performances for estimating seizure recurrence risk after routine EEG were validated in a temporally shifted cohort of 261 patients, where ROC AUC was 0.63, significantly above chance. In comparison, the presence of IEDs—the only validated marker of seizure risk in the clinical setting—was 0.61. A two-step model that uses first IEDs and then computational features on IED-negative EEGs achieved a testing AUC of 0.70. These performances were still significant with EEGs that did not capture any visible IEDs. The best performances were achieved in patients without focal lesion.

The most important finding of this study is the robust evidence for non-visible changes in the EEG signal associated with the propensity to have seizures. For decades, researchers have been hinting at non-visible differences in the EEG signal of PWE compared to healthy controls^13,37^. Two important frameworks to model the EEG are linear and non-linear models. Linear models assume that the signal arises from a linear combination of independent oscillators. In general, alpha frequency is found to be slower in patients with focal^22,38^ and generalized epilepsy^19^ Non-linear models represent the signal as a non-linear dynamical system that can be characterized by entropy and dimensionality, among others. Entropy and correlation dimension, both measures of signal complexity, tend to be reduced in PWE^23,24,39^. It must be emphasized that this body of literature is built upon small case–control studies^13^, a study design that overestimates diagnostic performances^40^.

In contrast to case–control designs, cohort or nested case–control studies reduce the risk of selection bias when evaluating diagnostic accuracy^41^. Two studies used this more robust approach to predict seizure recurrence from automated analysis of routine EEG^42,43^. In the first, the authors evaluated Paroxysmal slow wave events (PSWE, 2-s EEG windows with a median peak frequency of < 6 Hz) on a cohort of 70 patients undergoing EEG after a first seizure^42^. They found that the rate of PSWE could predict seizure recurrence at 18-month with an AUC of 0.72. In the second, on a cohort of 114 patients undergoing EEG after a first suspected seizure, the connectivity in the theta band could predict a future diagnosis of epilepsy with a specificity of 70% and sensitivity of 53%^43^. Importantly, neither study validated their findings on an separate set of patients. In our study, we adopted a cohort design: subjects were drawn consecutively from a population of patients undergoing routine EEG, i.e., the target population in a real-world setting. We also used a temporally shifted testing cohort, which allows to explore the out-of-sample generalizability of the models in a manner that mimics their real-life deployment. These factors reinforce the robustness of the performance estimation, which should be consistent when deployed in the clinical setting^44^. However, the testing cohort was from the same institution as the training set. The capacity of this method to generalize to other institutions would need to be evaluated in a future study.

While the correlation between seizure frequency and presence of IEDs on routine EEG is not well established, the ability to predict long-term seizure recurrence from routine EEG would impact both the management of patients presenting with suspected seizure(s) and patients diagnosed and treated for epilepsy. Currently, the prognostic value of EEG at diagnosis is mostly focused on the evaluation of patients with an unequivocal, single unprovoked seizure. In these patients, identification of IEDs on a single routine EEG confers a two-fold increase in the risk of subsequent seizures if untreated, generally warranting ASM therapy^45^. In addition, the prognostic value of EEG before ASM withdrawal is demonstrated in patients with at least two-year seizure freedom (especially in children)^46^. Beyond these clinical scenarios, there is still little evidence to support the use of EEG to adjust ASM therapy and prognosticate the disease since a highly active EEG does not necessarily correlate with seizure frequency; this restricts the usefulness of EEG as a monitoring tool^2,9,10,47^. In this study, we included all consecutive patients undergoing routine EEG at our institution; we are interested in the potential of the routine EEG to quantify at one point in time the propensity to have future seizures. When combined with IEDs in a two-step model, the algorithm showed a testing ROC AUC of 0.70. For context, in our cohort, IEDs alone had an AUC of 0.61. These results demonstrate a certain complementarity between EEG features and IEDs, and bring hope that the routine EEG could potentially be used as a tool to assist clinicians in recommending for or against ASM treatments based on future seizure risk. However, the usefulness and real-life impact of this algorithm would need to be established in a prospective clinical setting. Moreover, while ML-based analysis of EEG holds important promises, it will only ever serve as additional data to physicians, allowing them and their patients to make more informed decisions.

The best models for each outcome had a ROC AUC on the internal validation cohort of 0.67, 0.64, and 0.66 for seizure recurrence at one year, epilepsy diagnosis, and active epilepsy, respectively. While these performances are statistically significant, their clinical usefulness could be questioned, especially with regards to the limited PPV and specificity at a given threshold. Two major restrictions impede predictive performances: the capacity of the model and the reliability of the labels used for training. First, the small variance seen across different models and outcomes might indicate that the amount of data used saturated the capacity of the features and models (i.e.: underfitting). Machine learning studies consistently show that dataset size is correlated with predictive performances given sufficient capacity of the model^48,49^. The next step to improve performances would therefore consist in gathering more training data to increase the complexity of the EEG features and ML architecture. The second hurdle is the confidence in the labels. Epilepsy diagnosis is probabilistic by definition: a patient could be at “high risk” of seizure and therefore have epilepsy, but never go on to have seizure recurrence^1^. Nevertheless, the performances for this outcome were still above chance on the temporally shifted cohort. The models trained to predict the outcome “Active epilepsy” did not generalize well to the testing cohort. We used this outcome to test the hypothesis that having had recent seizures would strongly affect the EEG signal. The results indicate that the features that we extracted from the EEG signal are more affected by seizure propensity (risk of having seizure recurrence) than past seizures. Ultimately, all these outcomes depend on the imperfect reporting of seizures by patients^50^; there is promise that in the future, more objective outcomes could directly benefit predictive models in epilepsy^51^.

EEG signals are altered by age, sex, co-morbidities, antiseizure medications, and possibly several other factors; however, the degree to which these variables can confound predictions made from the EEG signal is unknown^52–55^. In our study, the models trained to predict seizure recurrence at one year were robust to clinical variables. Patients with a focal finding on neuroimaging had reduced predictive performance, but we did not observe worse performance in patients with focal slowing on EEG. There was a poor correlation of focal findings on neuroimaging to focal slowing on EEG: only 37% (72) of EEGs with focal findings on neuroimaging had focal slowing, and 38% (54/144) with focal slowing on EEG did not have a focal finding on neuroimaging. This poor correlation highlights that neuroimaging and EEG query different aspects of the nervous system. Further work is needed to improve performance of our algorithm in patients with underlying non-epileptic focal abnormalities by using spatially aware features (e.g. left–right or anterior–posterior gradients, topographical voltage maps)^56,57^ or connectivity features^58,59^. The post-hoc analyses were mostly limited by the number of patients, especially under the robust nested CV framework.

The survival analysis showed that ASMs count and age were important predictors of seizure recurrence risk. Higher ASMs count is a proxy for refractory cases and higher seizure frequency. ASMs are, however, infrequently accounted for in previous studies^60–62^. In our case, grouping by number of ASMs did not affect performances for prediction of seizure recurrence, suggesting that extracted features were independent of ASM. Performances for patients with no ASM could not be reliably estimated because of the rare seizure recurrence in this subgroup (11 EEGs). A small sample size also prevented the subgroup analysis for predicting the success of ASM withdrawal (32 EEGs). These two clinical applications would need to be explored in future studies. Regarding age, older patients carry a higher disease burden and are at higher risk of syncope, transient neurologic episodes, and confusion—nonepileptic conditions that could lead a patient to undergo an EEG exam. This partly explains why the yield of routine EEG for epilepsy in older patients is much lower^54^. Here, seizure recurrence was rare in the older patient group (> 60 y.o.), for which performance could not be estimated. As predicted, the addition of age as an input feature slightly improved predictions. The potential benefit of using age as a predictor may be limited by the resulting increase in dimensionality that could overthrow the increase in information, especially in a data-scarce setting that is subject to overfitting.

The model had above-chance performances in the absence of IEDs for all outcomes. Previously, only a few studies tested the impact of IEDs on their model; most had a case–control design, and none validated their results on a separate validation set^43,56,61,63^. This finding suggests that automated analysis of EEG would increase the yield of EEG even in the absence of IEDs (74.7% of all EEGs in our cohort). The low sensitivity of EEG for IEDs leads to delays in diagnosis and need for prolonged or repeated exam, so the “negative” subgroup of EEGs (without IEDs) would most directly benefit from an alternative and independent marker. The two-step classifier showed an increase in performance compared to IEDs or computational features alone. This could orient the clinical applications of such an algorithm, complementing the interpretation of the EEG reader when an EEG does not reveal IEDs. Recent studies have demonstrated that machine learning models can detect IEDs with expert-level performances^64,65^. Our approach could complement these algorithms by predicting seizure risk in IED-negative EEG, to further improve the clinical value and objectivity of EEG.

BP had the greatest performances when used alone to predict seizure recurrence, followed by FuzzEn and LL. Studies have suggested that slight shifts can be observed in the frequency spectrum of patients with epilepsy^19–21,66,67^. This could be secondary to the pathologically increased interictal synchronization, but could also be explained by ASM, age, or other confounders. In our study, compared with other features, BP had greater decrease in performances in younger patients and in the absence of abnormal slowing. Interestingly, it was the most performant feature in the presence of multiple ASMs (in opposition to FuzzEn and SampEn), suggesting that the changes in the frequency spectrum is not related to these confounders. Regarding entropy, it is generally found to be lower in patients with focal and generalized epilepsy^23,24^. Increased predictability and reduced complexity could result from the constraints imposed by epileptogenic processes^68^. Several algorithms have been proposed to estimate the entropy of physiological time-series, without clear evidence that this measure can embody seizure propensity^69^. Compared with other features, FuzzEn was more affected by the presence of IEDs, focal lesion, and in patients with focal epilepsy; other entropy markers followed a similar trend. Entropies also had poor performances in patients with ≥ 2 ASMs, in line with previous studies on non-linear analysis of EEG^70^.

This complementarity between BP and entropy could be leveraged with clinical priors in the modeling process.

Despite the methodological strengths of this study, some limitations must be highlighted. First, the data comes from a single center, preventing us to generalize the results to other institutions. Second, the data collection was retrospective. For some patients, follow-up might have been too short to detect seizure recurrence, and these patients would have been inappropriately flagged as “no seizure recurrence”. This limitation decreases the potential effect size (our capacity to discriminate between groups), and is, as such, conservative in nature. Third, most patients were on ASM at the time of EEG. ASM are known to affect the EEG signal and several of the features used in this study, such as BP and entropy^19,20,69^. A larger sample size is required to estimate the performances of such markers in patients with no ASM or in those undergoing ASM withdrawal. Finally, while statistically significant, the clinical impact of the performances reported in this study is modest. Applied to the clinical setting, the models would affect risk estimation by only a few percent, as demonstrated by the modest PPV, NPV, specificity, and sensitivity. This could potentially be addressed by using more powerful (albeit data-hungry) models to represent the EEG data, such as deep neural networks. With these limitations in mind, the findings in this study still robustly suggest that there exist changes in the EEG signal other than IEDs that can inform us about long-term seizure propensity; this opens the door to the possibility of using automated markers of epilepsy in the clinical setting, and strongly motivates future research in this direction.

In conclusion, we demonstrate that there are changes other than IEDs in the EEG signal embodying seizure propensity. These changes have a predictive horizon of one year after the EEG and their significance is independent of IEDs, age, and number of antiseizure medications. While significant, the potential impact on decision making in the clinical setting is modest. Future work will focus on improving the representation of the EEG to increase the performances of this approach and evaluate its real-life impact on clinical decision making.

### Supplementary Information

Supplementary Information 1.

Supplementary Information 2.

## Data Availability

All code written for this study will be publicly available upon publication at the following address: https://gitlab.com/chum-epilepsy/epilepsy_markers_reeg.git. Anonymized data in the form of extracted features with clinical outcomes will be provided upon reasonable request to the corresponding author and conditional to the approval by our ethics research board. The TRIPOD reporting checklist is provided as supplementary material.
