# Supplementary material for: Machine-learning for the prediction of one-year seizure recurrence based on routine electroencephalography
Source: Sci Rep. 2023 Aug 4;13:12650. doi: 10.1038/s41598-023-39799-8 (PMC10403587; doi:10.1038/s41598-023-39799-8)
Supplement: Supplementary file 1 — Supplementary Information 1. [file 41598_2023_39799_MOESM1_ESM.docx]

# Supplementary material

## Supplementary method 1: Algorithms for linear and non-linear features

| **Feature** | **Formula** | **Ref.** |
| --- | --- | --- |
| Band power | For the frequency range $\left( f_{1},f_{2} \right)$:  $\text{BP}\left( x, f_{1},f_{2} \right)=2\int_{f_{1}}^{f_{2}} S_{x}\left( f \right)df ,$  where $S_{x}$ is the power spectral density of a signal $x$. | ^1^ |
| Correlation dimension | For an embedding dimension $m$, the correlation dimension $D$ is described as:  $C\left( r \right)\sim r^{D} .$  $D$ is approximated by fitting the power law. The correlation integral $C\left( r \right)$ is estimated with the equation:  $\hat{C}\left( r,m \right)=\frac{1}{N\left( N-1 \right)}\sum_{i=1}^{N} \sum_{j!=i}^{N} \theta\left( r-D_{\mathrm{euclidian}}\left( x_{i}^{m},x_{j}^{m} \right) \right) ,$  where $\mathbf{x}_{i}$ is the time series of delay vector $\mathbf{x}_{i}^{m}=\left( x_{i-m+1},x_{i-m+2},\ldots,x_{i} \right)$ with embedding dimension $m$, and $\theta\left( x \right)$ is the Heaviside step function. | ^2^ |
| Line length | For a time-series of length $N$:  $L=\sum_{i=1}^{N} \left\vert x_{i+1}-x_{i} \right\vert.$ | ^3^ |
| Peak alpha frequency | The peak frequency for the range (8, 13 Hz) is:  $\text{PAF}\left( x \right)=\arg\max_{8, 13} S_{x}\left( f \right) ,$  where $S_{x}$ is the power spectral density of a signal $x$. | ^4^ |
| Hurst exponent | The Hurst exponent $H$ is defined as:  $\mathbb{E}\left[ \frac{R\left( n \right)}{S\left( n \right)} \right]=Cn^{H} as n \to\infty,$  where $\mathbb{E}\left[ \frac{R\left( n \right)}{S\left( n \right)} \right]$ is the averaged rescaled range over all partial time series of length $n$. $H$ is obtained by fitting the equation $\log\left( \frac{R\left( n \right)}{S\left( n \right)} \right)=H\log\left( n \right)+C$ with the least-squared methods. | ^5^ |
| Approximate entropy | For a length of pattern $m$ and tolerance $r$:  $\text{ApEn}\left( x,m,r \right)=\phi^{m}\left( r \right)-\phi^{m+1}\left( r \right)$  with  $\phi^{m}\left( r \right)=-\frac{1}{N-m+1}\sum_{i=1}^{N-m+1} \log C_{i}^{m}\left( r \right) ,$  where $N$ is the length of the signal, $C_{i}^{m}\left( r \right)$ is the number of counts of $j$ where $D_{\text{Chebyshev}}\left( x_{i},x_{j} \right)\leq r$ for all subsamples in $x_{i,j}^{m},\ldots,x_{N}^{m}$. | ^6^ |
| Sample entropy | For a length of pattern $m$ and tolerance $r$:  $\text{SampEn}\left( x,m,r \right)=-\log\frac{\sum_{i=1}^{N-m} A_{m}^{i}\left( r \right)}{\sum_{i=1}^{N-m} B_{m}^{i}\left( r \right)} ,$  where $N$ is the length of the signal, $A_{i}^{m}\left( r \right)$ is the number of counts where $D_{\text{Chebyshev}}\left( x_{i},x_{j} \right)\leq r$ for all subsamples in $x_{i,j}^{m+1},\ldots,x_{N}^{m+1}$, and $B_{i}^{m}\left( r \right)$ is the number of counts where $D_{\text{Chebyshev}}\left( x_{i},x_{j} \right)\leq r$ for all subsamples in $x_{i,j}^{m},\ldots,x_{N}^{m}$, excluding self-counts ($j=i$). | ^7^ |
| Fuzzy entropy | Same as Sample entropy, with the counts $A_{m}^{i}$ and $B_{m}^{i}$ replaced by the fuzzy membership function:  $D\left( x_{i},x_{j} \right)=\exp\left( \frac{-D_{\text{Chebyshev}}\left( x_{i},x_{j} \right)^{n}}{r} \right) .$ | ^8^ |
| Permutation entropy | For a permutation order $k$:  $\text{PermEn}\left( x,k \right)=-\sum p\left( \pi_{k} \right)\log p\left( \pi_{k} \right) ,$where $p\left( \pi_{k} \right)$ is the relative frequency of the permutation pattern $\pi_{k}$. | ^9^ |
| Spectral entropy | For the frequency range $\left( f_{1},f_{2} \right)$:  $\text{SpecEn}\left( x,f_{1},f_{2} \right)=-\sum_{f_{i}=f_{1}}^{f2} p_{f_{i}}\log\left( \frac{1}{p_{f_{i}}} \right) ,$where $p_{f}$ is the power spectral density at frequency $f$. | ^10^ |
|  | | |

## Supplementary Table S1: Reasons for ordering index EEG in the internal validation cohort.

|  | Number of EEGs |
| --- | --- |
| Follow-up EEG | 186 |
| Characterization of epilepsy | 53 |
| Pre-withdrawal | 32 |
| Post epilepsy surgery | 13 |
| New seizure type | 9 |
| Not yet diagnosed |  |
| Transient neurological deficit | 74 |
| Altered mental status | 60 |
| Syncope | 33 |
| Abnormal movements | 33 |
| Single unprovoked seizure | 27 |
| Other | 29 |

## Figure S1: Directed acyclic graph for the selection of covariates in the survival analysis


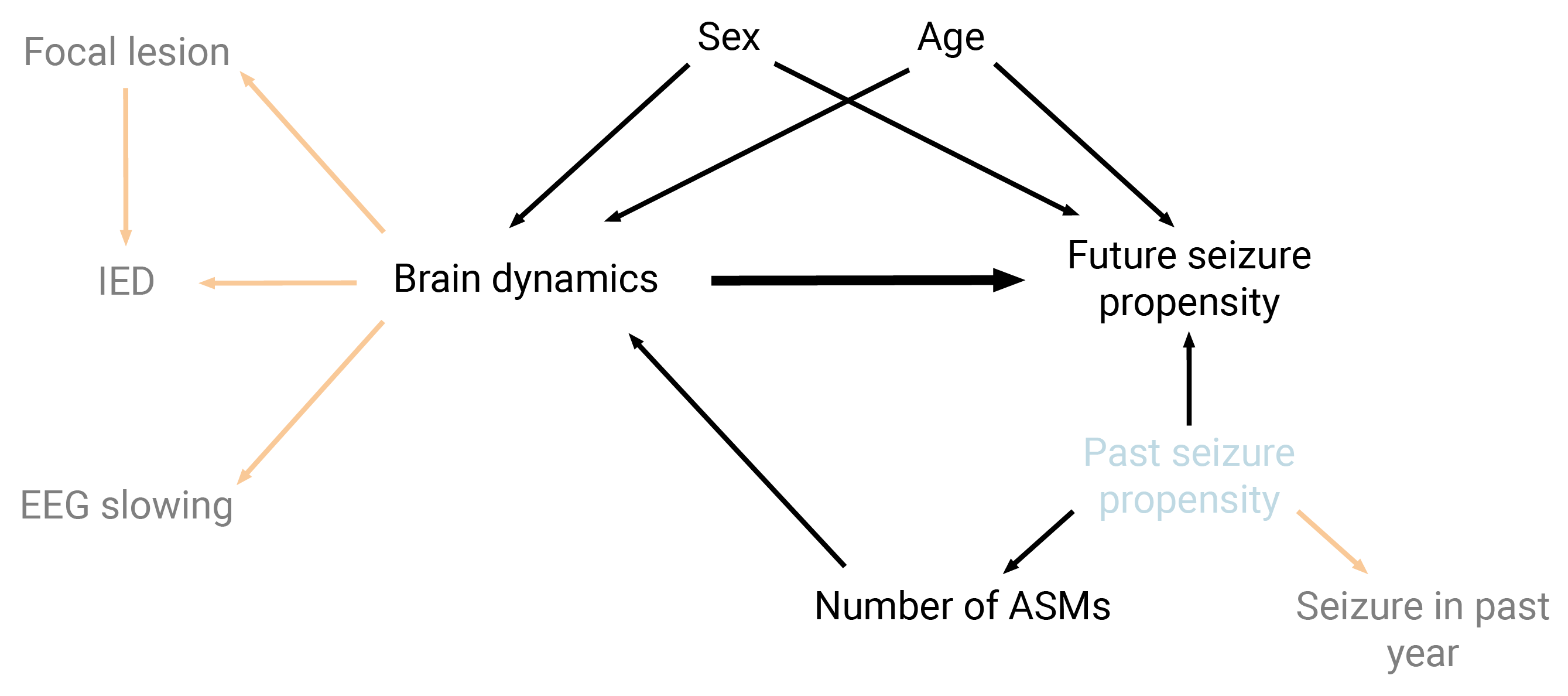


**Figure S1**: Directed acyclic graph for survival model. Black labels: Minimal sufficient adjustement set for estimating the total effect of Brain dynamics (EEG features) on Future seizure propensity (risk of seizure recurrence). Grey labels: Variables that do not impact causal pathway. Blue label: unobserved variable. Bold black arrow: causal path. Thin black arrows: biasing paths. Orange arrows: non-biasing paths. ASM: antiseizure medication; IED: interictal epileptiform discharge.

## Figure S2: Subgroup analysis for outcomes “epilepsy diagnosis” and “epilepsy active pre-EEG”


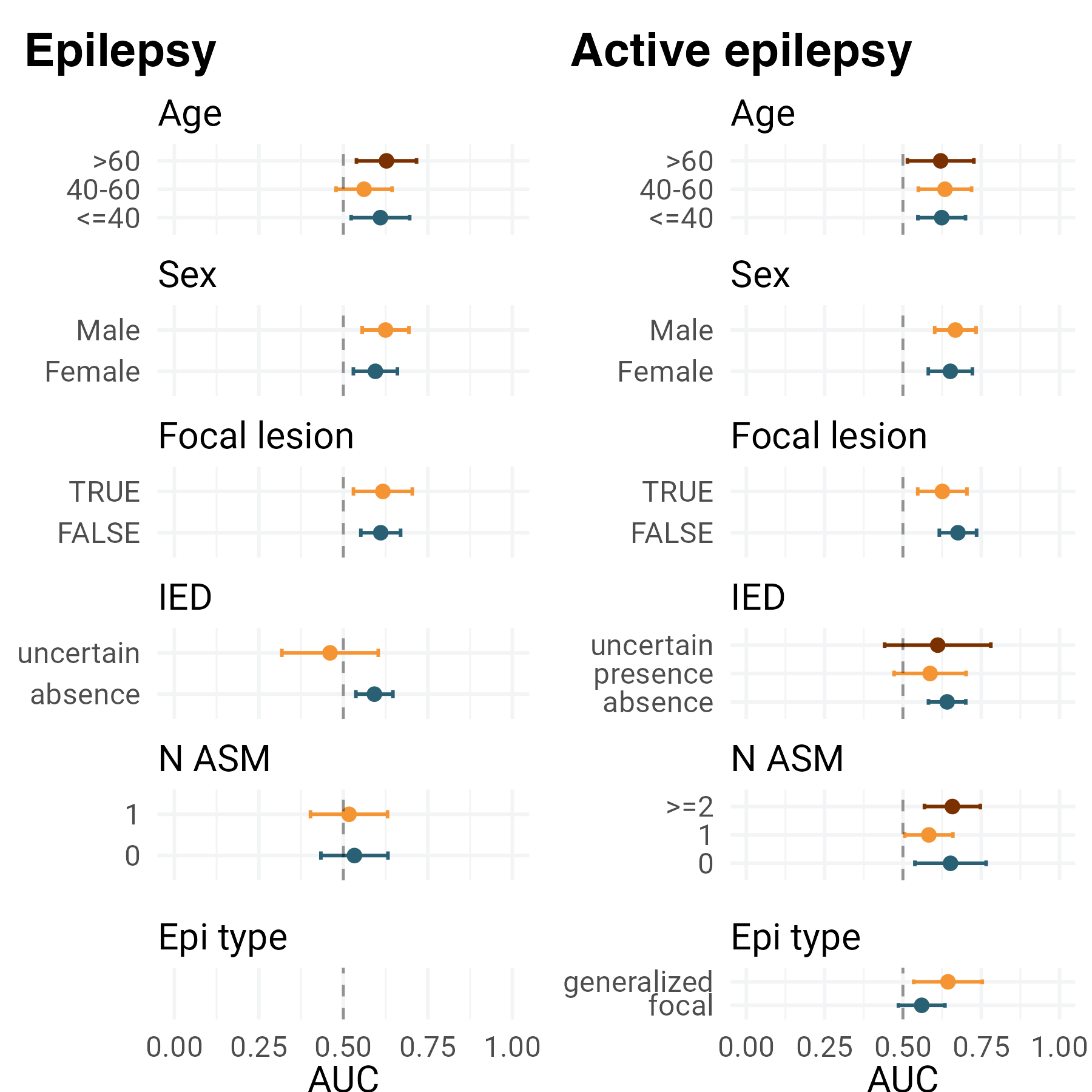


**Figure S2**: Predictive performances (ROC AUC) for the LightGBM model stratified by subgroups for the outcomes “Epilepsy” and “Active epilepsy”, with 95% confidence intervals. The dotted line indicates AUC of 0.50. AUC ROC: Area-under-the-receiver operating characteristic curve.

## Figure S3: Subgroup analysis for each individual marker


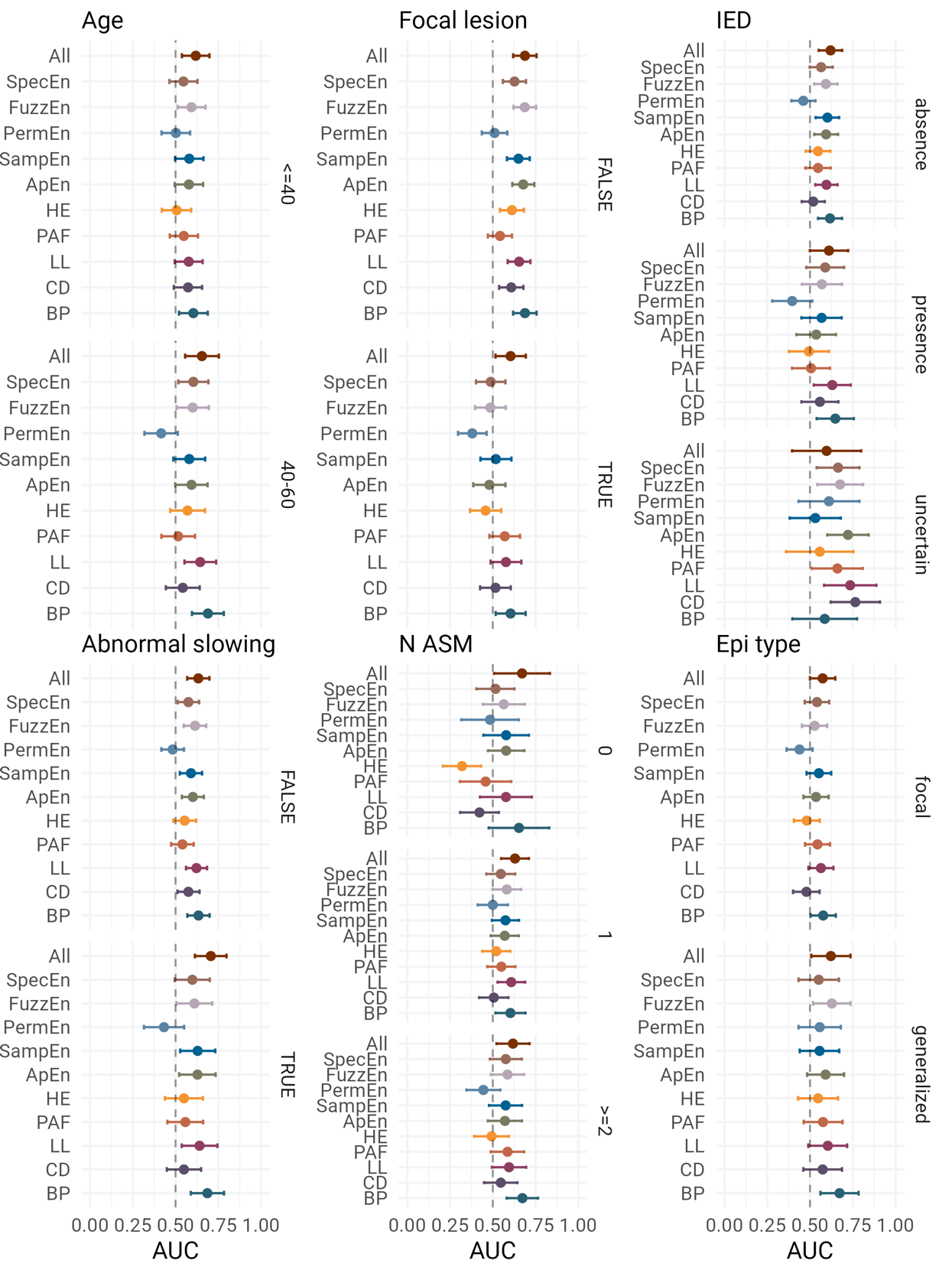


**Figure S3:** Predictive performances (ROC AUC) for the LightGBM model stratified by subgroups for each of the three outcomes, with 95% confidence intervals. The dotted line indicates AUC of 0.50. AUC ROC: Area-under-the-receiver operating characteristic curve.

## Table S2: Model AUC for each outcome for different percentiles used to aggregate predictions of the epochs from a single EEG. The results are drawn from the internal validation cohort via nested Cross-validation. In bold: highest value for an outcome-classifier pair; in italics: lowest value.

| **Outcome** | **Classifier** | **0.1** | **0.2** | **0.3** | **0.4** | **0.5** | **0.6** | **0.7** | **0.8** | **0.9** |
| --- | --- | --- | --- | --- | --- | --- | --- | --- | --- | --- |
| Seizure recurrence | **GLM** | *0.588* | 0.593 | 0.597 | 0.601 | 0.599 | 0.603 | 0.605 | 0.602 | **0.606** |
| Seizure recurrence | **LGBM** | **0.635** | 0.624 | 0.625 | 0.626 | 0.625 | 0.628 | 0.625 | *0.617* | 0.621 |
| Seizure recurrence | **RF** | **0.651** | 0.642 | 0.640 | 0.638 | 0.635 | 0.637 | 0.635 | *0.627* | 0.630 |
| Seizure recurrence | **SVM** | 0.608 | 0.618 | **0.622** | **0.622** | 0.620 | 0.618 | 0.614 | 0.610 | *0.607* |
| Epilepsy | **GLM** | 0.623 | 0.629 | 0.631 | 0.629 | **0.633** | 0.629 | 0.631 | 0.629 | *0.619* |
| Epilepsy | **LGBM** | 0.626 | **0.628** | 0.623 | 0.618 | 0.618 | 0.612 | 0.613 | 0.610 | *0.608* |
| Epilepsy | **RF** | *0.615* | 0.619 | 0.618 | 0.620 | 0.621 | **0.627** | 0.625 | 0.623 | 0.617 |
| Epilepsy | **SVM** | 0.636 | 0.634 | 0.635 | 0.635 | **0.642** | 0.638 | 0.632 | 0.625 | *0.616* |
| Active epilepsy | **GLM** | **0.592** | 0.585 | 0.580 | 0.582 | 0.580 | 0.586 | 0.587 | *0.575* | 0.579 |
| Active epilepsy | **LGBM** | 0.638 | 0.637 | 0.643 | 0.649 | **0.653** | 0.648 | 0.644 | 0.637 | *0.626* |
| Active epilepsy | **RF** | **0.667** | 0.664 | 0.663 | 0.664 | 0.663 | 0.661 | 0.660 | *0.649* | 0.653 |
| Active epilepsy | **SVM** | **0.589** | 0.584 | 0.583 | *0.579* | 0.581 | 0.583 | 0.585 | 0.585 | 0.580 |

##

## Supplementary method 2: Sensitivity analyses for survival model

We performed a sensitivity analysis for the survival analysis. We repeated the multivariate survival analyses with different control variables to estimate the variation in the adjusted Hazards Ratio (aHR) of seizure recurrence according to the model’s predictions under alternative DAG models. The analysis would be robust to the causal assumptions if all aHR estimates were statistically significant.

| Covariates set | Adjusted hazard ratio for the model’s prediction impact on seizure free survival (95%CI) |
| --- | --- |
| Model predictions | 1.44 (1.24–1.66) |
| Model predictions + gender | 1.43 (1.24–1.66) |
| Model predictions + gender + sex | 1.24 (1.06–1.46) |
| Model predictions + gender + sex + N ASM | 1.18 (1.01–1.28) |
| Model predictions + gender + sex + N ASM + focal lesion | 1.18 (1.01–1.38) |
| Model predictions + gender + sex + N ASM + focal lesion + abnormal slowing | 1.21 (1.03–1.42) |

## Supplementary method 3: Sample size estimation

Power analysis was performed using the R library *powerMediation*, according to Hsieh et al. (1998).^11^ We estimated a range of sample sizes for a logistic regression with the binary predictor being the class predicted by the model: 0 for low-predicted risk and 1 for high-predicted risk. We assumed a base rate of outcome of 0.4. We use a significance level of 0.05 / 12 (four models $\times$ three outcomes) and target power of 0.9. The odds ratio (OR) is the increased rate of outcome when the model predicts high risk of outcome. For an OR of 1.5, the required sample size is 425 per group. For an OR of 1.75, 2.0, and 2.25, the sample sizes are 184, 98, and 57 per group, respectively. Therefore, with an average of 1 200 EEGs per year and an exclusion rate of 0.3 (included EEGs ≈ 840), we estimated that all routine EEGs from a single year would provide us with sufficient statistical power.

## References

1. Cohen, M. X. *Analyzing Neural Time Series Data: Theory and Practice*. (2014). doi:10.7551/mitpress/9609.001.0001.

2. Grassberger, P. & Procaccia, I. Measuring the strangeness of strange attractors. *Physica D: Nonlinear Phenomena* **9**, 189–208 (1983).

3. Esteller, R., Echauz, J., Tcheng, T., Litt, B. & Pless, B. Line length: an efficient feature for seizure onset detection. in *2001 Conference Proceedings of the 23rd Annual International Conference of the IEEE Engineering in Medicine and Biology Society* vol. 2 1707–1710 vol.2 (2001).

4. Gelety, T. J., Burgess, R. J., Drake, M. E., Jr., Ford, C. E. & Brown, M. E. Computerized spectral analysis of the interictal EEG in epilepsy. *Clinical Electroencephalography* **16**, 94–97 (1985).

5. Luo, K. & Luo, D. An EEG feature-based diagnosis model for epilepsy. in *2010 International Conference on Computer Application and System Modeling (ICCASM 2010)* vol. 8 V8-592-V8-594 (2010).

6. Pincus, S. M. Approximate entropy as a measure of system complexity. *Proceedings of the National Academy of Sciences* **88**, 2297–2301 (1991).

7. Richman, J. S. & Moorman, J. R. Physiological time-series analysis using approximate entropy and sample entropy. *Am J Physiol Heart Circ Physiol* **278**, H2039-2049 (2000).

8. Chen, W., Wang, Z., Xie, H. & Yu, W. Characterization of Surface EMG Signal Based on Fuzzy Entropy. *IEEE Transactions on Neural Systems and Rehabilitation Engineering* **15**, 266–272 (2007).

9. Bandt, C. & Pompe, B. Permutation Entropy: A Natural Complexity Measure for Time Series. *Phys. Rev. Lett.* **88**, 174102 (2002).

10. Inouye, T. *et al.* Quantification of EEG irregularity by use of the entropy of the power spectrum. *Electroencephalogr Clin Neurophysiol* **79**, 204–210 (1991).

11. Hsieh, F. Y., Bloch, D. A. & Larsen, M. D. A simple method of sample size calculation for linear and logistic regression. *Stat Med* **17**, 1623–1634 (1998).
